# MicroRNA-410-5p exacerbates high-fat diet-induced cardiac remodeling in mice in an endocrine fashion

**DOI:** 10.1038/s41598-018-26646-4

**Published:** 2018-06-08

**Authors:** Tong Zou, Mei Zhu, Yi-Cheng Ma, Fei Xiao, Xue Yu, Li Xu, Lan-Qing Ma, Jiefu Yang, Jian-Zeng Dong

**Affiliations:** 1 Department of Cardiology, Anzhen Hospital, Capital Medical University, Beijing, China; 2 Department of Cardiology, Beijing Hospital, Ministry of Health, Beijing, China; 3 Department of Ultrasound, The First Affiliated Hospital, Kunming Medical University, Kunming, China; 4 State Key Laboratory for Conservation and Utilization of Bio-Resources in Yunnan, Yunnan University, Kunming, China; 5 Yunnan Institute of Digestive Disease, Department of Digestive Diseases, First Affiliated Hospital, Kunming Medical University, Kunming, Yunnan China

## Abstract

Metabolic disorders, such as obesity and type 2 diabetes, are associated with an increased risk of cardiomyopathy. To date, microRNA (miRNAs) functions in cardiac remodeling induced by obesity remain to be elucidated. We found that rats fed a high fat diet (HFD) manifested cardiac fibrosis and LV dysfunction. In the heart of rats fed HFD, the phosphorylation levels of Smad 2 and the expression of fibrotic genes, such as connective tissue growth factor, collagen-1α1 (Col1α1), Col3α1, and Col4α1, were up-regulated, which accompanied by an increase in Smad 7 protein levels, but not its mRNA levels. Using miRNA microarray analysis, we showed that the miRNA miR-410-5p inhibited the protein expression of Smad 7, thus increasing the phosphorylation levels of Smad 2. Overexpression of miR-410-5p promoted cardiac fibrosis in rats fed normal diet, whereas inhibition of miR-410-5p by way of miR-410-5p antimiR suppressed cardiac fibrosis in rats fed HFD. Finally, our data revealed that miR-410-5p from the kidney and adipose tissues was probably transferred to heart to induce cardiac fibrosis. Taken together, our study characterizes an endocrine mechanism in which adipose- or kidney-derived circulating miR-410-5p regulates metabolic disorders-mediated cardiac remodeling by activating the TGFβ/Smad signaling in heart.

## Introduction

Obesity is a global health problem contributing to development of various metabolic-related disease states, including type 2 diabetes and dyslipidemia. Furthermore, obesity is strongly associated with an increased risk for hypertension, myocardial infarction, endothelial dysfunction and coronary artery disease^1^. Excessive accumulation of lipids in the heart also causes cardiac remodeling, which is illustrated by the histologic characteristics, such as cardiac fibrosis, myocardial hypertrophy, and lipotoxic cardiomyopathy^2–5^.

The molecular mechanisms underlying obesity-cardiac remodeling are not fully understood. Previous studies have demonstrated that oxidative stress and inflammation are associated with cardiac fibrosis in obese mice^2,6,7^. Furthermore, transforming growth factor β (TGFβ) signaling pathway plays a major role in the development of cardiac fibrosis in rats with obesity, diabetes, and hypertension^6,8,9^. TGFβ1 interacts with its receptor, a complex of trans-membrane serine/threonine kinase receptors (TGFβRI/TGFβRII), leading to phosphorylation of Smads 2/3, the R-Samds. The activated Smad 2/3 then bind to the Co-Smad, Smad 4. This trimeric complex is translocated from cytoplasm into nuclei to regulate transcription of fibrotic genes^10,11^. Regulation of the TGFβ signaling occurs at several levels. For instance, inhibitory Smads (I-Samds), such as Smad 7, antagonizes the TGFβ signaling by blocking the association of the activated Smad 2/3 with Smad 4 or competitively inhibiting R-Smad phosphorylation by TGFβRI. Meanwhile, Smad 7 recruits E3 ubiquitin ligases and their co-factors to TGFβ ligand/receptor complexes, targeting the complexes for degradation. In addition, a large number of E3 ligases regulate the stability of these three types of Smads by polyubiquitination and proteasomal degradation. The TGFβ signaling regulates a set of fibrotic genes, thereby eliciting the fibrogenesis by increasing collagen deposition and extracellular matrix expansion. Inhibition of TGFβRI by its inhibitor markedly suppresses left ventricular remodeling in rat models with myocardial infarction^12^. However, how the TGFβ signaling is activated in obesity is not fully understood. It has been shown that the expression of TGFβ1 and the phosphorylated levels of Smad 2/3 are significantly up-regulated in myocardial tissues of mice or rats fed high fat diet (HFD)^6,8^. In contrast, Aubin *et al*. have reported that the amount of the TGFβ1 transcript is not altered in rats fed HFD^13^.

MicroRNAs (miRNAs) are a large class of endogenous, small non-coding RNAs of approximately 22 nucleotides in length. miRNAs mainly target specific mRNAs through imperfect base pairing with the 3′-untranslated region (3′UTR) of these mRNAs, leading to either translational repression or degradation of the target mRNAs^14^. miRNAs are involved in a variety of cardiac diseases, including pulmonary arterial hypertension, cardiac hypertrophy, and heart failure^15–19^. Recent studies have demonstrated that miRNAs also play a role in obesity-induced cardiac pathophysiology^20,21^. miR-451 is involved in diabetic cardiomyopathy, such as cardiac hypertrophy through suppression of the LKB1/AMPK pathway in mice fed HFD^20^. miR-322 that upregulated in leptin-deficient obese (ob/ob) mice has a cardioprotective effect by modulating the insulin pathway^21^. It is likely that many other miRNAs are involved in the regulation of cardiomyopathy in obesity. However, miRNA functions in cardiac fibrosis induced by obesity remain to be elucidated.

To better understand the role of miRNAs in cardiac fibrosis, we used microarray analysis to identify differentially regulated miRNAs in the heart of rats fed HFD. We focused on miR-410-5p, which was markedly up-regulated in the cardiac tissues of rats fed HFD. We found that miR-410-5p activated the TGFβ signaling pathway by targeting Smad 7. Finally, our data showed that miR-410-5p from the kidney and adipose tissues probably induced cardiac fibrosis.

## Results

### High fat diet induces cardiac fibrosis and alters cardiac function

**Figure 1:**
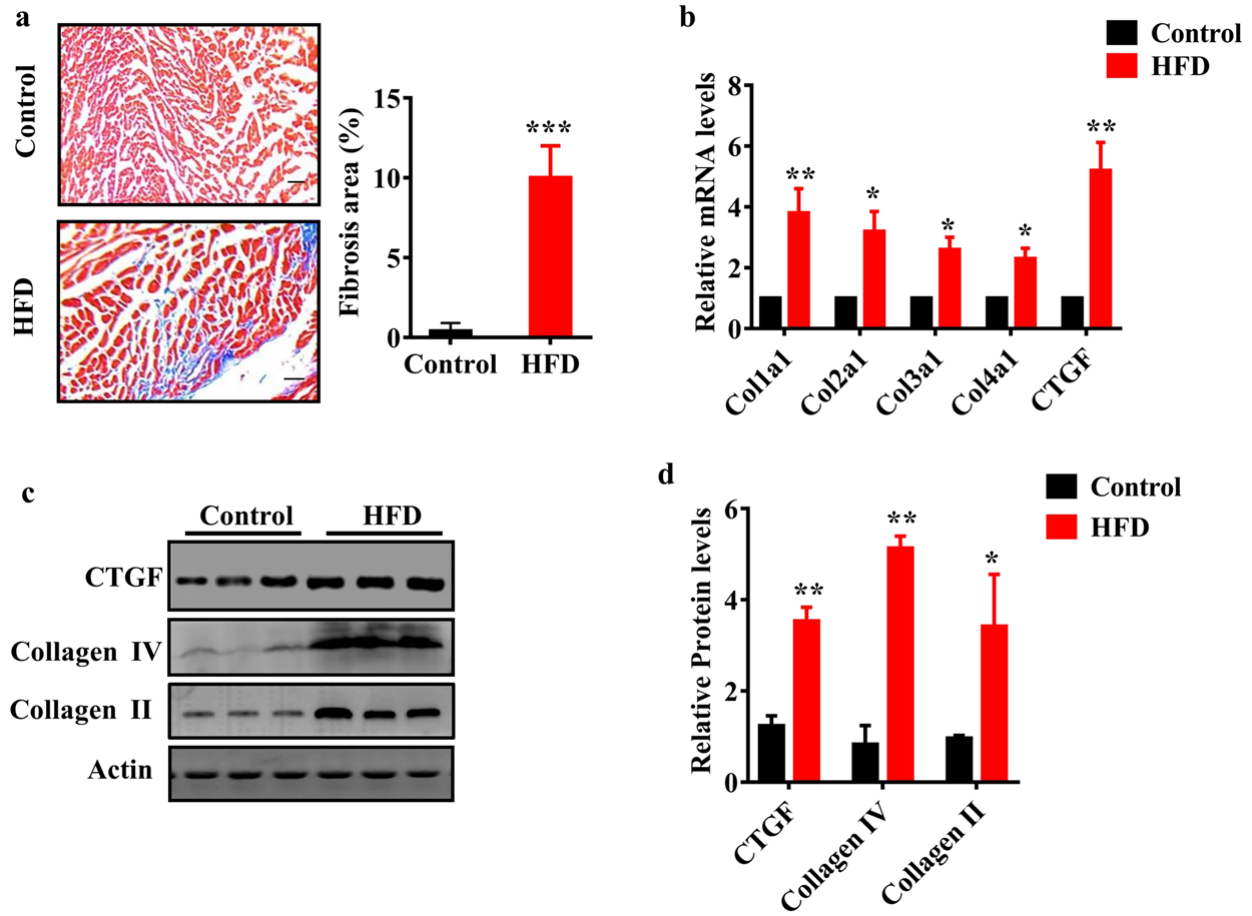
Cardiac fibrosis and expression of fibrotic markers in the heart of rats fed HFD. (**a**) Cardiac matrix deposition in Masson-stained sections of rats fed HFD and control rats. Heart tissues from the HFD group show focal regions of fibrosis in the interstitium (Left panels). Quantitative analysis of fibrotic area (Right panel). These results are means ± SD of four experiments (n = 10 in each experiment). ***P < 0.001 relative to control (normal group). Scale bar, 20 μm. (**b**) The mRNA levels of fibrotic genes in the heart of rats were detected by qPCR. All results are standardized to the levels of GADPH and the means ± SD of three experiments (n = 10 in each experiment). *P < 0.05; **P < 0.01 relative to control (normal group). (**c**) The levels of fibrotic proteins were measured using Western blotting (n = 6 in each experiment). Representative Western blots are shown. (**d**) Quantification of the ratio of proteins to β-actin. These results are the means ± SD of three experiments. *P < 0.05; **P < 0.01 relative to control (normal group).

Rats became markedly obese, with significant increases in body weight after 24 weeks of HFD feeding (Table 1). We examined the myocardial fibrosis in the left atria using Masson trichrome staining. As shown in Fig. 1a, HFD caused a marked deposition of collagens. Next, we found that HFD notably up-regulated the mRNA expressions of fibrotic markers, such as connective tissue growth factor (CTGF), collagen-1α1 (Col1α1), Col3α1, and Col4α1 (Fig. 1b). Similarly, the protein levels of CTGF, collagen-II, and collagen-IV were also up-regulated in the heart of rats fed HFD (Fig. 1c,d).

**Table 1:** The body weight, liver function and lipid metabolism in rats fed HFD.

	Control	HFD	*P*-value
Body weight (g)	420 ± 54	536 ± 63	<0.05
Alanine aminotransferase (U/L)	47.5 ± 15.6	92.4 ± 17.8	<0.05
Aspartate aminotransferase (U/L)	93.6 ± 30.6	346.7 ± 45.5	<0.05
Total cholesterol (mmol/L)	1.72 ± 0.40	6.08 ± 2.51	<0.05
Free cholesterol (mmol/L)	0.29 ± 0.09	1.65 ± 0.78	<0.05
LDL cholesterol (mmol/L)	0.68 ± 0.07	1.85 ± 0.58	<0.05
HDL cholesterol (mmol/L)	0.81 ± 0.11	0.36 ± 0.09	<0.05
Triglyceride (mmol/L)	0.41 ± 0.08	0.92 ± 0.12	<0.05

**Figure 2:**
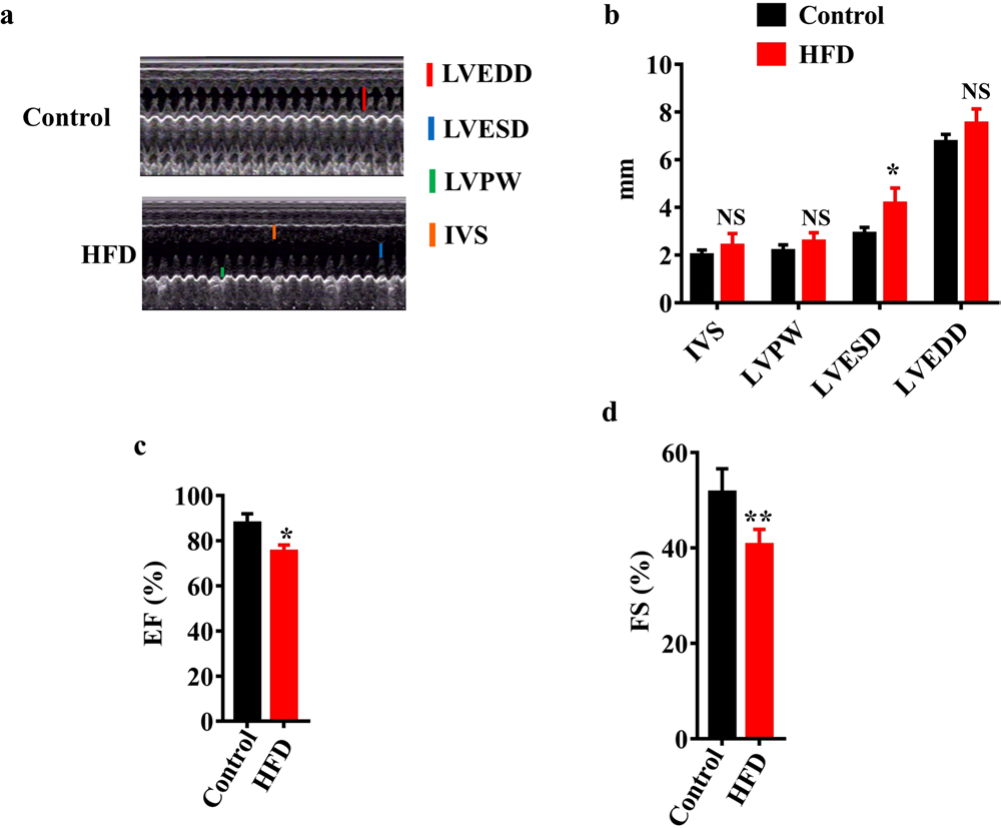
HFD impairs LV function. (**a**) M-mode echocardiogram. Comparison of general appearance of rats fed HFD (lower panel) and control rats (upper panel). The measurement of interventricular septum (LVS) and LV posterior wall (LVPW), LV end-systolic diameter (LVESD), and LV end-diastolic diameter (LVEDD) is shown. (**b**) LVS, LVPW, LVESD, and LVEDD. (**c**) LV ejection fraction (EF). (**d**) LV fractional shortening^23^. These results are means ± SD of three experiments (n = 10 in each experiment). *P < 0.05; **P < 0.01 relative to control (normal group).

As cardiac fibrosis are associated with left ventricular (LV) remodeling^22^, we performed echocardiography to evaluate the effect of HFD on LV dysfunction. M-mode standard two-dimensional (2DE) left parasternal-long axis echocardiographic examination was conducted (Fig. 2a). The echocardiographic findings showed that the thickness of interventricular septum (IVS) and LV posterior wall (LVPW) were comparable in HFD and control rats (Fig. 2b). Whereas the LV end-systolic diameter (LVESD) was significantly higher in rats fed HFD than in control rats, the LV end-diastolic diameter (LVEDD) did not differ between these two groups (Fig. 2b). Conversely, LV ejection fraction (EF) and the LV fractional shortening (FS)^23^ in rats fed HFD were significantly lower than those of the control rats (Fig. 2c,d). These results suggest that HFD impairs LV function.

### TGF-β1 pathway is activated by HFD

**Figure 3:**
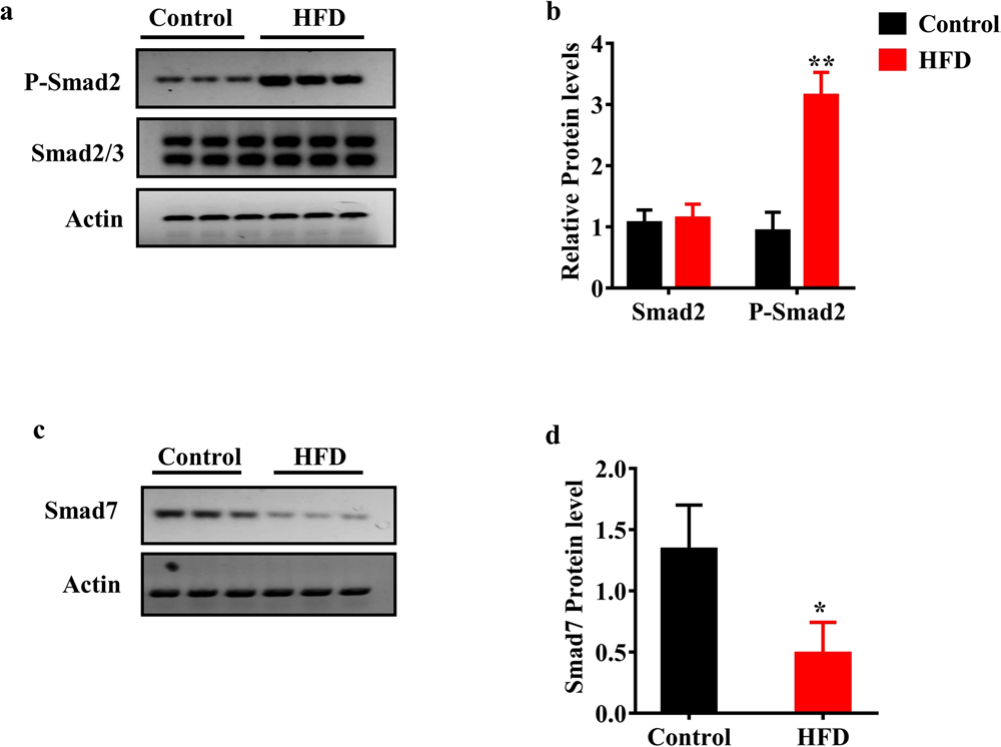
The TGFβ signaling is activated in the heart of rats fed HFD. (**a**) The levels of phosphorylated Smad 2 were increased in the heart of rats fed HFD (n = 6 in each experiment). Representative Western blots are shown. (**b**) Quantification of the ratio of proteins to β-actin. These results are the means ± SD of three experiments. **P < 0.01 relative to control (normal group). (**c**) The protein levels of Smad 7 were reduced in the heart of rats fed HFD (n = 6 in each experiment). (**d**) Quantification of the ratio of proteins to β-actin. These results are the means ± SD of three experiments. *P < 0.05 relative to control (normal group).

As the TGFβ signaling plays an important role in myocardial fibrosis, we tested the phosphorylation of Smad 2, an indicator of the activation of the TGFβ pathway. We found that the phosphorylation levels of Smad 2 were markedly increased in the heart of rats fed HFD (Fig. 3a,b), suggesting HFD-induced activation of the TGFβ pathway. The activation of the TGFβ pathway is probably due to an increase in expression of TGFβ1 or a decrease in expression of Smad 7, a key negative regulator of the TGFβ pathway. However, we found that the mRNA levels of TGFβ1 and Smad 7 were comparable in the heart of rats fed HFD and control rats (Fig. S1). When the protein expression of Smad 7 was analyzed, we found that the protein levels of Smad 7 were significantly down-regulated in the heart of rats fed HFD (Fig. 3c,d). These results implicate that HFD activates the TGFβ signaling, at least in part, by inhibiting the protein expression of Smad 7.

### miR-410 is up-regulated by high fat diet and suppresses Smad 7 translation

**Figure 4:**
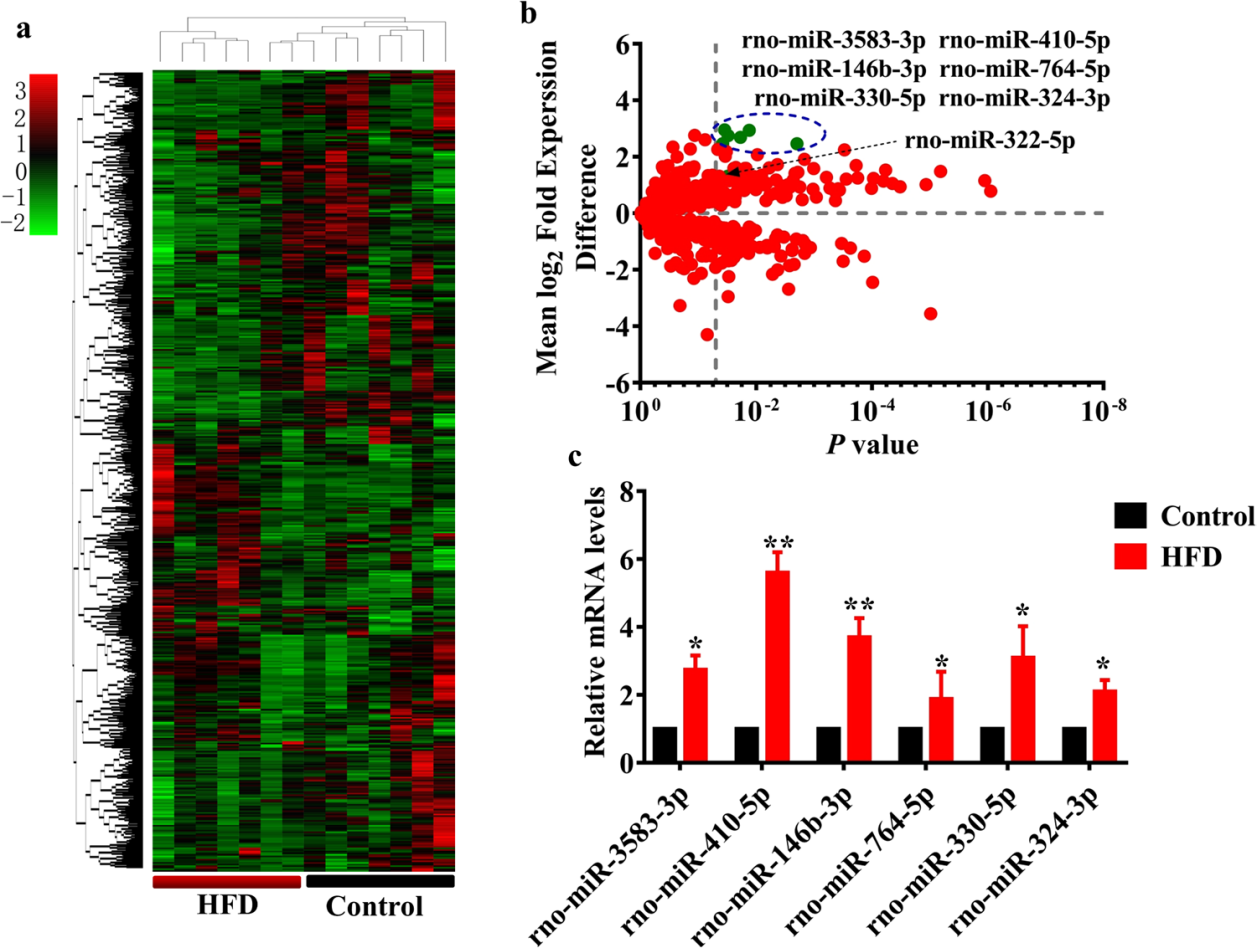
The miRNA expression profiles in the heart of rats. (**a**) Heat maps show significantly up- and down-regulated mRNAs in the heart of rats fed HFD and control rats for 24 weeks (n = 7 in each experiment). miRNA expression profiles were clustered hierarchically. (**b**) Volcano Plot for differential miRNA expression in the heart of rats. The green dost represent the most significantly upregulated miRNAs (more than five-fold, *P* < 0.05), including miR-3583-3p, -410-5p, -146b-3p, -764-5p, -330-5p, and -324-3p, in the heart of rats fed HFD for 24 weeks. The arrow indicates miR-322-5p. (**c**) qPCR analysis of the six most-up-regulated miRNAs in the heart of rats. All results are standardized to the levels of 18S rRNA and the means ± SD of three experiments (n = 6 in each experiment). *P < 0.05; **P < 0.01 relative to control (normal group).

**Figure 5:**
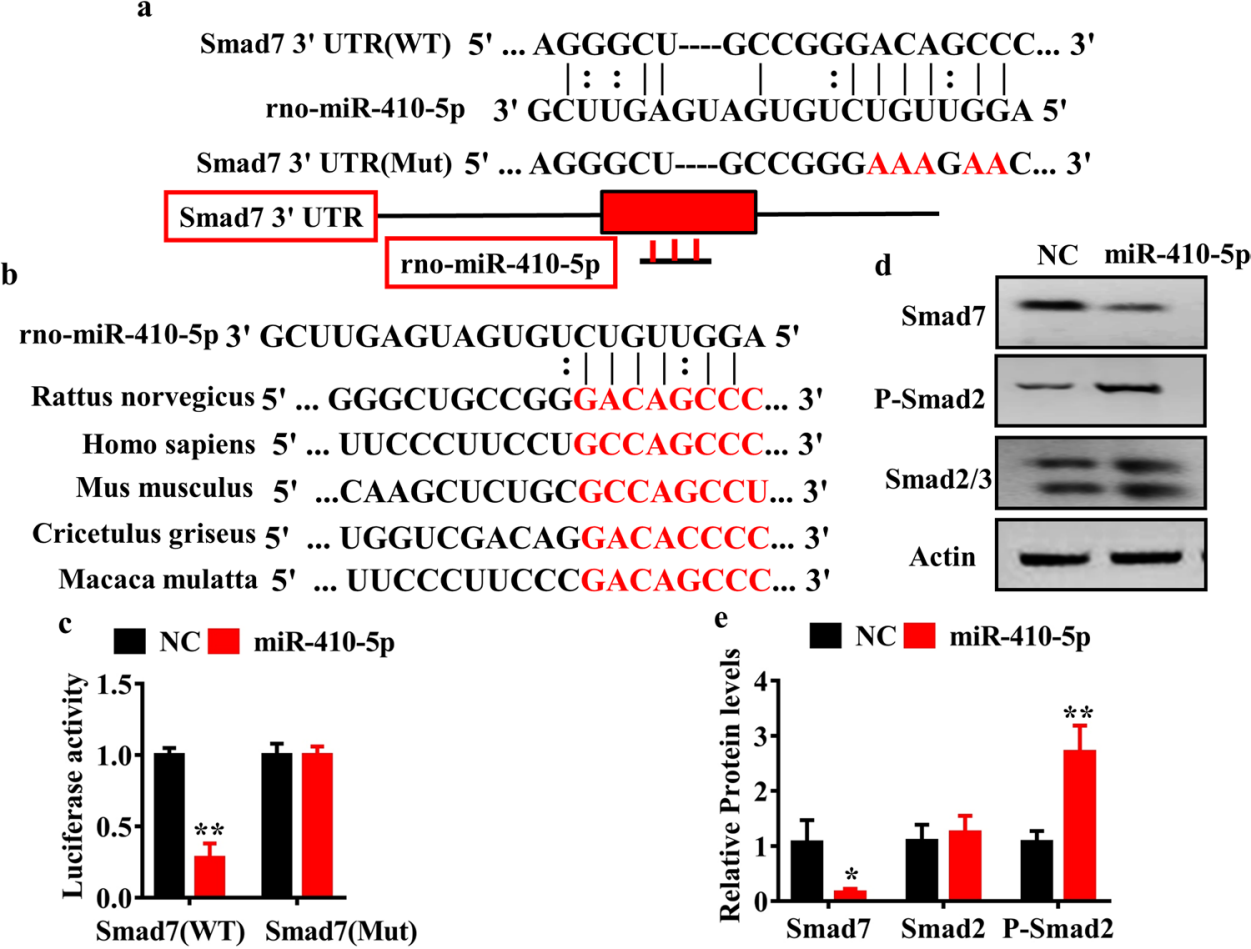
Smad 7 is a target gene of miR-410-5p. (**a**) Complementarity between the 3′UTR of Smad 7 gene and miR-410-5p. For constructing of Smad 7-3′UTR (Mut) reporter, the putative miR-410-5p binding site was replaced with the sequence as shown below (Mut). Mutated seeds were marked in red, Watson-Crick base pairing with a straight line and U-G wobbles with a dotted line. (**b**) The putative target sites of Smad 7 3′-UTR, which are marked in red, are conserved in mammals. (**c**) Luciferase analysis of a reporter vector harboring the 3′-UTR of Smad 7 (WT and Mut) in H9c2 cells transfected with negative control (NC) or miR-410-5p for 36 h. These results are means ± SD of three experiments. **P < 0.01 relative to NC. (**d**) Western blot analysis in H9c2 cells transfected with NC or miR-410-5p for 36 h. Representative Western blots are shown. (**e**) Quantification of the ratio of proteins to β-actin. These results are the means ± SD of four experiments. *P < 0.05; **P < 0.01 relative to NC.

The discrepant expression of Smad 7 transcript and protein suggest that HFD likely inhibits the expression of Smad 7 by a post-transcriptional mechanism. Since miRNA is a widely known mechanism for such regulation, we investigated whether miRNAs were involved in the suppression of Smad 7 protein. We first determined the miRNA expression profiles using miRNA microarray in the heart of rats fed HFD for 24 weeks (Table S1). We found that miR-322-5p was one of these up-regulated miRNAs (greater than 2-fold changes) (Fig. 4a,b). The miRNA, which protects against cardiac dysfunction, is also upregulated in ob/ob mice^21^. Our data revealed that miR-3583-3p, -410-5p, -146b-3p, -764-5p, -330-5p, and -324-3p were the most significantly upregulated miRNAs (more than five-fold, P < 0.05) in the heart of rats fed HFD (Fig. 4a,b). Quantitative real-time PCR (qRT-PCR) assay showed that the expression of these miRNAs was increased significantly (Fig. 4c). Of the six most up-regulated miRNAs, we identified miR-410-5p that could bind to the 3′UTR of Smad 7 by using a miRNA target prediction algorithm (RNA22) (Fig. 5a). Importantly, miR-410-5p is conserved miRNA among many species including human and rodents (Fig. 5b). Thus, miR-410-5p represents a promising candidate to regulate Smad 7.

To elucidate the involvement of miR-410-5p in the regulation of Smad 7 expression, we construct a vector that contained wild-type of the Smad 7-3′-UTR of or a 5-bp mutation in the miR-410-5p seed sequence recognition site for luciferase reporter assays (Fig. 5a). The luciferase activity of Smad 7-3′-UTR was markedly inhibited in H9c2 cells after transfection of miR-410-5p (Fig. 5c). In contrast, the mutation abrogated the reduction of the luciferase activity in response to miR-410-5p (Fig. 5c). To test whether miR-410-5p regulated Smad 7 endogenously, we determined the effect of miR-410-5p overexpression on Smad 7 expression in cardiocyte cell line H9c2. Although overexpression of miR-410-5p did not obviously influence the mRNA expression of Smad 7 (Fig. S2), it markedly suppressed the protein levels of Smad 7 (Fig. 5d,e). Meanwhile, overexpression of miR-410-5p significantly promoted the phosphorylated levels of Smad 2 (Fig. 5d,e). These results suggest that miR-410-5p targets Smad 7 for translational inhibition.

### miR-410-5p promotes cardiac fibrosis

**Figure 6:**
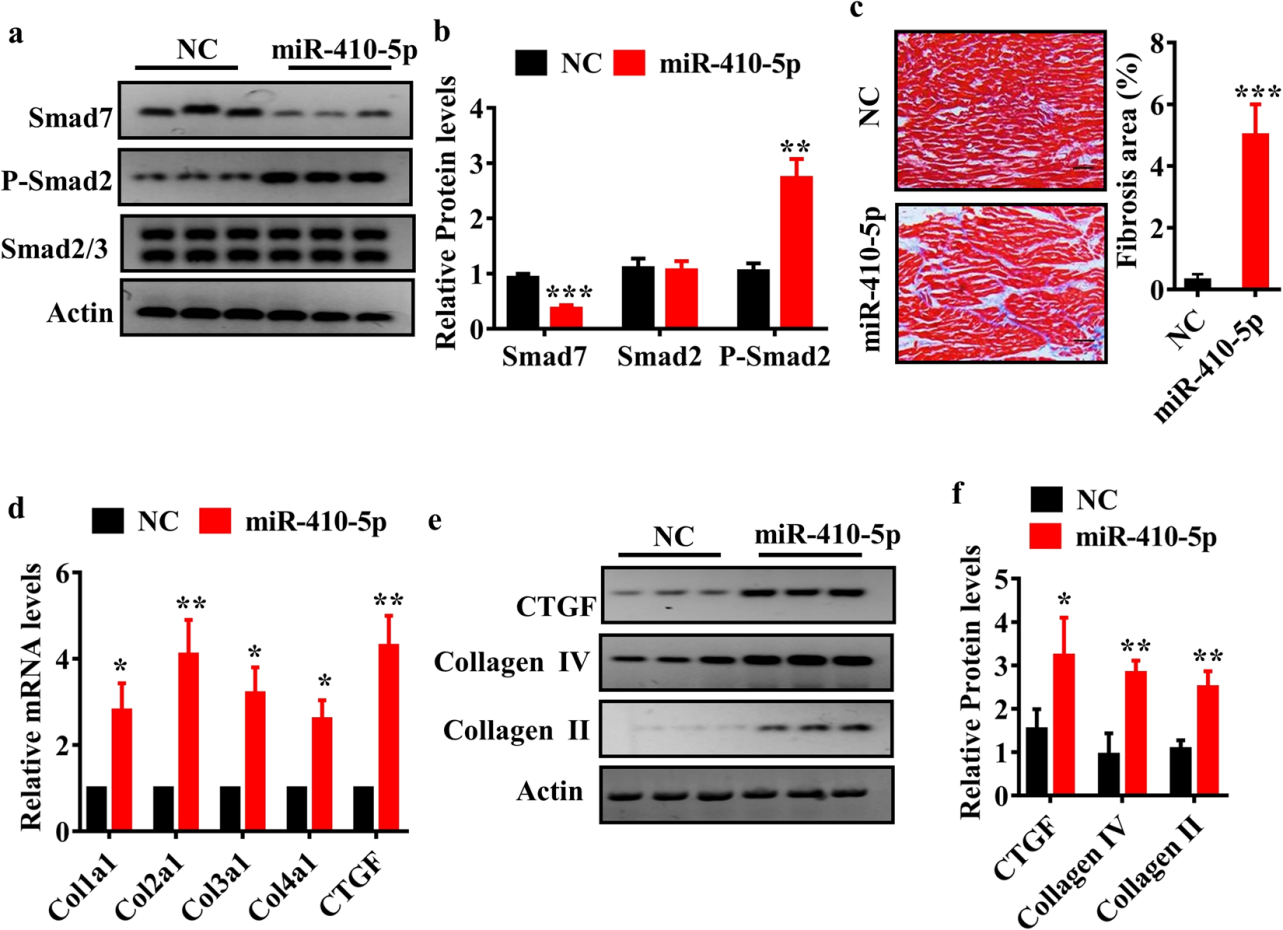
Overexpression of miR-410-5p promotes cardiac fibrosis. The rats were infected with lentivirus particles containing PGLV3-miR-410 or PGLV3-NC. After 21 days injection, animals were euthanized for further analysis. (**a**) The protein levels of genes in the TGFβ pathway were measured using Western blotting (n = 6 in each experiment). Representative Western blots are shown. (**b**) Quantification of the ratio of proteins to β-actin. These results are the means ± SD of three experiments. **P < 0.01; ***P < 0.001 relative to negative control (NC). (**c**) Masson’s staining of heart in rats. Heart tissues from miR-410-5p overexpression group showed focal regions of fibrosis in the interstitium (Left panels). Quantitative analysis of fibrotic area (Right panel). These results are means ± SD of three experiments (n = 10 in each experiment). ***P < 0.001 relative to NC. Scale bar, 20 μm. (**d**) The mRNA levels of fibrotic genes in the heart of rats were detected by qPCR. All results are standardized to the levels of GAPDH and the means ± SD of three experiments (n = 6 in each experiment). *P < 0.05 relative to NC. (**e**) The protein levels of fibrotic genes were measured using Western blotting (n = 6 in each experiment). Representative Western blots are shown. (**f**) Quantification of the ratio of proteins to β-actin. These results are the means ± SD of three experiments. *P < 0.05; **P < 0.01 relative to NC.

To investigate the effect of elevated miR-410-5p expression on cardiac fibrosis, miR-410-5p was overexpressed in the whole body via lentiviral vector (PGLV3-miR-410). As expected, injection of rats with PGLV3-miR-410 led to a decrease in the protein levels of Smad 7 and an increase in the phosphorylation levels of Smad 2 (Fig. 6a,b). Furthermore, formation of collagen fibers was detected by Masson’s trichrome staining in the heart of miR-410-5p-treated rats compared with control rats (Fig. 6c). Next, we determined the mRNA levels of CTGF, Col1α1, Col3α1, and Col4α1 by qPCR and found significant up-regulation of all four gene transcripts in the heart of miR-410-5p-treated rats (Fig. 6d). Similarly, the protein levels of CTGF, collagen-II, and collagen-IV were also increased in miR-410-5p-treated heart (Fig. 6e,f). Next, we tested the effect of antimiR knockdown of miR-410-5p on HFD-induced cardiac fibrosis. After fed HFD for 21 weeks, the rats were injected once weekly for three consecutive weeks with miR-410-5p antimiR. We found that miR-410-5p antimiR attenuated cardiac fibrosis, and restored the protein levels of Smad 7 in rats fed HFD (Fig. S3a,b). Together, these results suggest that miR-410-5p promotes cardiac fibrosis.

### miR-410-5p from the kidney and adipose tissues probably induces cardiac fibrosis

**Figure 7:**
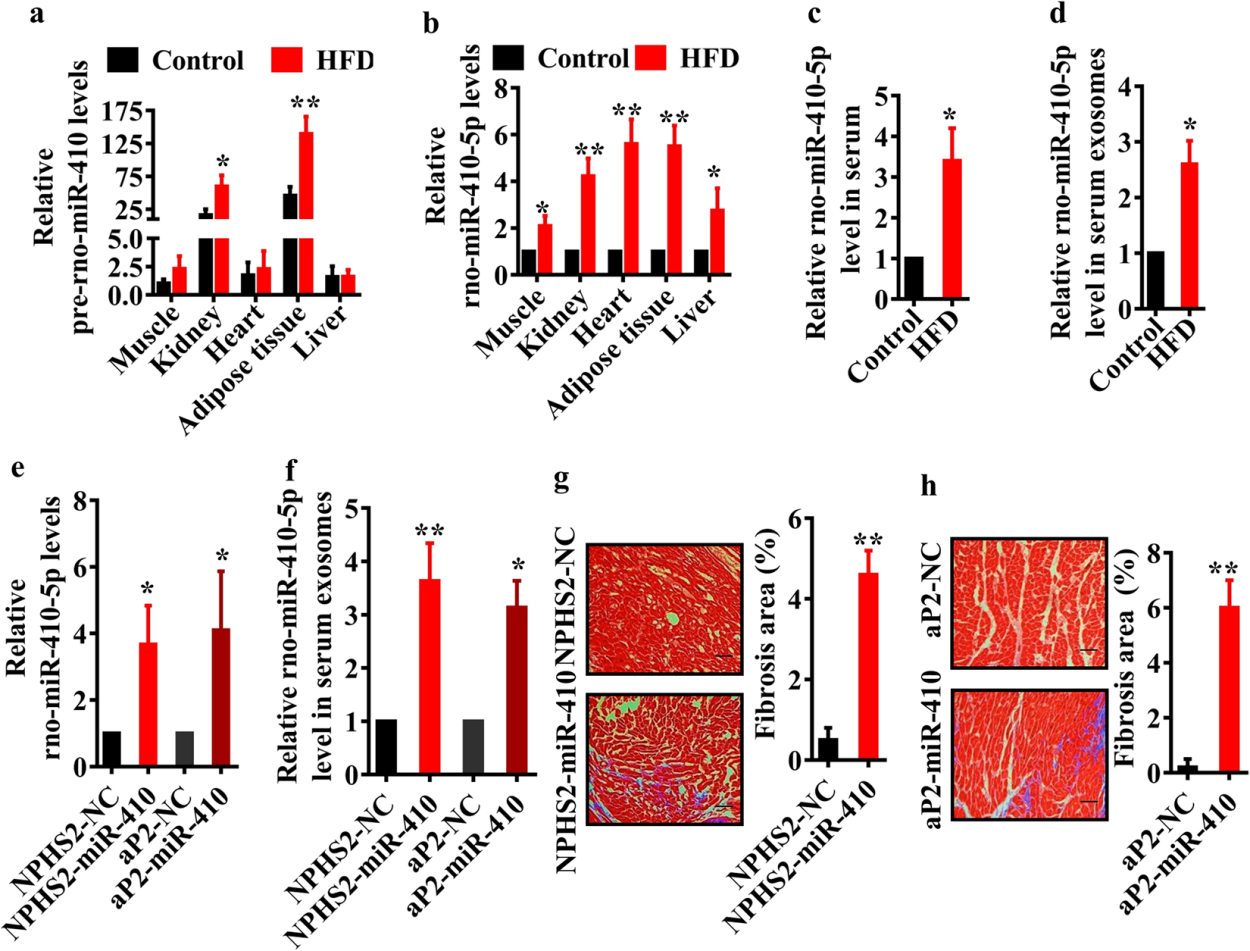
miR-410-5p from the kidney and adipose tissues promotes cardiac fibrosis. (**a**) The levels of pre-miR-410 (**a**) and miR-410-5p (**b**) were determined by qPCR in different tissues of rats fed normal diet and HFD, respectively. All results are standardized to the levels of 18S rRNA and the means ± SD of three experiments (n = 10 in each experiment). *P < 0.05; **P < 0.01 relative to control (normal group). (**c**,**d**) The levels of miR-410-5p in serum (**c**) and exosomes (**d**) of rats fed normal diet and HFD. These results are the means ± SD of three experiments (n = 10 in each experiment). *P < 0.05 relative to control. (**e**) The levels of miR-410-5p in the heart of rats fed normal diet with kidney- or adipocyte-specific overexpression of miR-410. NPHS2-miR-410, kidney-specific overexpression; aP2-miR-410, adipocyte-specific overexpression. *P < 0.05 relative to negative control (NC). All results are standardized to the levels of 18S rRNA and the means ± SD of three experiments (n = 10 in each experiment). (**f**) The levels of miR-410-5p in exosomes of rats fed normal diet with kidney- or adipocyte-specific overexpression of miR-410. These results are the means ± SD of three experiments (n = 10 in each experiment). *P < 0.05; **P < 0.01 relative to NC. (**g**,**h**) Masson’s staining of heart in rats (Left panels). Quantitative analysis of fibrotic area (Right panel). These results are means ± SD of three experiments (n = 10 in each experiment). **P < 0.01 relative to NC. Scale bar, 20 μm.

When searching the tissue distribution of miR-410-5p in database^24^, we found that miR-410-5p is only expressed in renal and adipose tissues. To confirm this point, we detected pre-miR-410 in major organs, such as liver, heart, kidney, muscle and adipose tissues using qPCR. Our results revealed that the levels of pre-miR-410 in the kidney and adipose tissues were 16~40-fold higher than those in the liver, heart, and muscle tissues (Fig. 7a). Furthermore, the expression of pre-miR-410 was significantly up-regulated only in the kidney and adipose tissues, but not in other tissues, after HFD feeding. Interestingly, the levels of miR-410-5p were significantly increased in all the tissues of rats fed HFD, compared to those in control rats (Fig. 7b). As the expression of pre-miR-410 in the heart, liver, and muscle was much lower than those in the kidney and adipocyte, the elevated miR-410-5p levels in these tissues were probably derived from those in circulation released from renal and adipose tissues. To test this hypothesis, we first examined the levels of miR-410-5p in the serum, and found that the miR-410-5p levels were notably elevated in the serum of rats fed HFD (Fig. 7c). It is believed that a large fraction of circulating miRNAs exist in in exosomes (<100 nm vesicles that are released from multivesicular bodies)^25^. We thus isolated exosomes from serum and determined the levels of miR-410-5p using qPCR. We found that the levels of miR-410-5p in exosomes of rats fed HFD were markedly higher than those in control rats (Fig. 7d). Finally, we tested the effect of kidney- or adipose-specific overexpression of miR-410-5p on cardiac fibrosis. As shown in Fig. S4a, tissue-specific overexpression of miR-410-5p led to a significant increase in miR-410-5p expression in kidney or adipose tissue. Meanwhile, we found that overexpression of miR-410-5p in either kidney or adipose increased the levels of miR-410-5p in the heart and exosomes (Fig. 7e,f). Overexpression of miR-410-5p in both tissues induced cardiac fibrosis (Fig. 7g,h), which were accompanied with reduced protein levels of Smad 7 (Fig. S4b–d). Therefore, it is likely that miR-410-5p that expressed in the renal and adipose tissues is secreted into the bloodstream, and then transferred to the heart to induce cardiac fibrosis.

## Discussion

**Figure 8:**
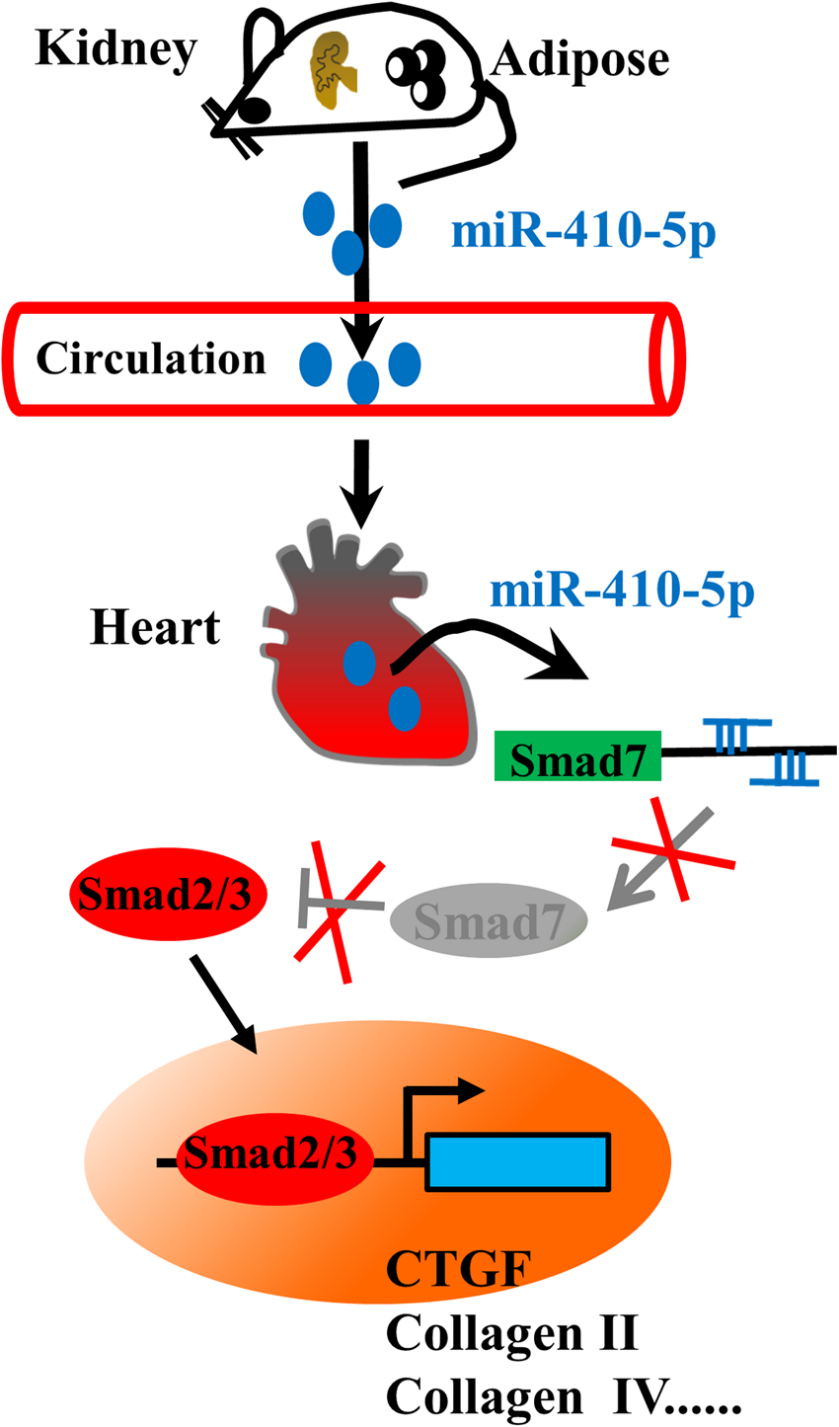
A proposed mechanism of miR-410-5p-induced cardiac fibrosis in rats with obesity.

To date, it has been well-established that metabolic disorders due to overnutrition, such as obesity, promote cardiac remodeling. However, the role of miRNAs in cardiac remodeling induced by obesity remains unknown. In this report, we have demonstrated that miR-410-5p is involved in cardiac fibrosis, an important part of cardiac remodeling, in rats fed HFD. miR-410-5p probably derived from the kidney or adipose tissues negatively regulates the protein expression of Smad-7 in the heart through direct base pairing to the 3′ UTR of its mRNA (Fig. 8). Suppression of Smad 7 activates the TGFβ signaling pathway, which in turn elicits cardiac fibrosis. Thus, our study characterizes an endocrine mechanism in which miR-410-5p regulates metabolic disorders-mediated cardiac remodeling.

In this study, biochemical and histological analysis have revealed that compared with normal controls, cardiac fibrosis is associated with increases in the expression of fibrotic genes, such as CTGF, Col1α1, Col3α1, and Col4α1, in the heart of obese rats. Meanwhile, echocardiographic examination has further demonstrated that LVEF and LV fractional shortening are significantly lower, whereas LVESD, an index of LV remodeling, is markedly higher in obese animals. Our results are consistent with previous studies observations that long-term feeding of HFD to rodents results in cardiac fibrosis and LV dysfunction^2,3,5,7,13,26–28^.

In general, mature miRNAs are derived from both the 5′ and 3′ arms of a pre-miRNA hairpin, termed the 5p-miRNA and 3p-miRNA^29^. In this study, miR-410-5p is identified by miRNA microarray analysis and the miRNA target prediction algorithm RNA22. miR-410 belongs the largest known mammalian miRNA cluster, the Gtl2-Dio3 noncoding RNA locus^30^. Two recent studies have link miR-410-3p to cardiac diseases^31,32^. Clark *et al*. have demonstrated that miR-410-3p is significantly upregulated in the cardiac disease models of mice, including myocardial infarction and chronic angiotensin II stimulation, and in the cardiomyopathies associated with muscular dystrophies^31^. Inhibition of miR-410-3p in stressed cardiomyocytes attenuates the hypertrophic response. The up-regulation of miR-410-3p is also observed in a myocardial ischemia/reperfusion injury mice model and in hypoxia/reoxygenation-treated cultured human adult cardiac myocytes^32^. Overexpression of miR-410-3p induces apoptosis by targeting high-mobility group box 1 protein in cardiac myocytes. Unlike miR-410-3p, little is known about the physiological function of miR-410-5p. A recent study from Wang *et al*. has demonstrated that circulating miR-410-5p levels are significantly elevated in the patients with prostate cancer^33^. ROC curve analysis has shown that miR-410-5p was a specific diagnostic biomarker of prostate cancer. More recently, these authors have reported that the miR-410-5p can degrade the miR-410-3p through base pairing to form a duplex between the two miRNAs in DCs^34^. As miR-410-3p functions as an oncogene or tumor suppressor gene in some malignancies^35^, the degradation of miR-410-3p favors the tumor progression. Our data indicate that the expression of miR-410-5p is significantly up-regulated in the heart of rats fed HFD. Our *in vitro* and *in vivo* experiments demonstrate that miR-410-5p down-regulates the protein levels of Smad 7, resulting in activation the TGFβ signaling pathway and induction of fibrotic genes. Furthermore, overexpression of miR-410-5p in rats elicits cardiac fibrosis. Thus, obesity promotes cardiac fibrosis, at least in part, by regulating the miR-410-5p/TGFβ signaling. It should be noted that although the mRNA levels of TGFβ1 were unchanged in the heart of rats fed HFD, our data cannot exclude a role of TGFβ1 itself in cardiac fibrosis. It is very likely that circulating levels of TGFβ1 are elevated in animals with obesity and activates the TGFβ signaling pathway in the heart.

Our results reveal that the levels of miR-410-5p are markedly higher in exosomes of rats fed HFD than in control rats. The circulating miRNAs have been found within vesicles (eg. microvesicles and exosome) and proteins, such as Argonaute 2 (AGO2), high-density lipoproteins (HDL), and other RNA-binding proteins^36^. Exosomes, a class of small extracellular vesicles, can promote cell-cell communication by shuttling molecules, such as miRNAs between cells^37,38^. Recently, Thomou *et al*. have demonstrated that adipose tissue is an important source of circulating exosomal miRNAs, and these adipose-derived circulating miRNAs regulate whole-body metabolism and mRNA translation in other tissues, such as in the liver^39^. Our results demonstrate that the expression of pre-miR-410 in the kidney and adipose tissues are much higher than that in the heart. It has been shown that miR-410-3p is highly expressed in the perinatal heart and sharply decreased in the adult heart, suggesting a role in perinatal cardiac function^30^. Although the expression of miR-410-3p is induced in the cardiac disease models of mice^31,32^, the expression of pre-miR-410 is not significantly altered in the heart of rats after HFD feeding. In contrast, the expression of pre-miR-410 is markedly up-regulated in the kidney and adipose tissues of rats fed HFD. More importantly, overexpression of miR-410-5p in either kidney or adipose increases the levels of miR-410-5p in the heart and exosomes. Therefore, it is likely that the expression of miR-410-5p is up-regulated in the donor tissues, kidney and adipose in obese rats, and secreted into the bloodstream by packing into exosomes or binding to AGO2. The exosomal miR-410-5p is then transferred to the recipient tissue heart. Inhibition of Smad 7 protein expression by miR-410-5p leads to the activation of the TGFβ signaling, induction of fibrotic genes, and subsequent cardiac fibrosis.

In summary, our findings reveal that miR-410-5p acts as an endocrine regulator to promote metabolic disorders-mediated cardiac remodeling. miR-410-5p up-regulated in the kidney and adipose of obese rats is released into circulation. The kidney- or adipose-derived circulating miR-410-5p down-regulates the protein expression of its target Smad 7 in the heart, resulting in the activation of the TGFβ signaling. The TGFβ pathway, in turn, induces the expression of fibrotic genes, thereby promoting cardiac fibrosis.

## Materials and Methods

### Animals

Male Sprague–Dawley (SD) rats (12 weeks old, 220–240 g) SD rats were obtained from the Animal Center, Kunming Medical University (Kunming City, Yunnan, China). These animals were housed at a constant temperature of 20–22 °C, with a 12-hour light/dark cycle. All animal procedures conform to the Guide for the Care and Use of Laboratory Animals that was published by the US National Institute of Health (NIH Publication No. 8523, revised 1985). The study was approved by the Animal Care and Use Committee of Anzhen Hospital, Capital Medical University. All experimental protocol including any relevant details was approved by the Animal Care and Use Committee of Anzhen Hospital, Capital Medical University. The methods were carried out in accordance with the approved guidelines.

Rats were randomly divided into two groups, with 15 rats in each group. Group A served as a control group and was maintained on normal rat chow diet (SPF-03 grade; Keaoxieli Food Company, Beijing, China) throughout the experiment (24 weeks). The normal diet in our study was composed of 26% protein, 57% carbohydrate, and 5% fat. Rats in Groups B were fed a HFD containing 60% basic diet, 20% pork fat, 15% refined sugar, 1.5% cholesterol, 0.1% sodium cholate, 3.4% peanuts throughout the experiment (24 weeks). The diet was composed of 15.6% protein, 49.2% carbohydrate, and 24.5% fat.

Rats were sacrificed at the end of experiment. Serum and organs including heart, liver, kidney, adipose, and muscle were collected. A part of the tissues including heart was quickly frozen at −80 °C for further use. The remaining part of heart was used for histopathological examination.

### Histopathological examination

Fresh heart samples were collected. All the samples were fixed in 4% paraformaldehyde, embedded in paraffin, stained with hematoxylin and eosin (H&E) or Masson’s trichrome. The sections were examined under a light microscope and photographed with digital camera (Olympus). In addition, a color image analysis system (ImageJ) was used to calculate the ratio of the fibrotic area to the whole area.

### Measurement of serum biochemical parameters

Serum levels of ALT, AST, CHOL, FCHOL, LDL HDL, and TG were determined by an automated biochemistry analyzer (Hitachi 7060, Japan).

### Isolation of exosomes

Exosomes from serum were isolated and purified by ExoQuick™-TC Exosome Precipitation Solution (SBI, Mountain View, CA) according to manufacturer’s instructions. Briefly, serum (500 μl) was centrifuged at 3000 × g for 15 min to remove cells and cell debris. The supernatant was transferred to a sterile tube and incubated with 126 μl of ExoQuick™ Exosome Precipitation Solution overnight at 4 °C. After centrifugation at 1500 × g for 30 min, the supernatant was removed carefully by aspiration. The exosomes appeared as a white pellet at the bottom of the tube. The exosome pellet was resuspended in 1/10 of original volume using sterile PBS.

### Quantitative real-time RT-PCR (qPCR)

For measurement of mRNA levels, total RNA from tissue samples was isolated using RNAprep Pure Tissue kit (Tiangen, Beijing, China) according to the manufacturer’s instructions. Random-primed cDNAs were generated by reverse transcription of total RNA with TIANScript M-MLV (Tiangen). A real-time PCR analysis was performed with the ABI Prism 7000 Sequence Detection system (Applied Biosystems) using SYBR® Premix-Ex TagTM (Takara, Dalian, Chnia). GAPDH was used for an internal control. Primer sequences for these mRNAs were listed in Table S2.

Mature miRNAs from tissue samples, serum and exosomes were extracted using miRcute miRNA Isolation kit (Tiangen). Pre-miRNAs from tissue samples were extracted using RNAprep Pure Tissue kit (Tiangen). poly(A) modification and first-strand cDNA synthesis were performed with miRcute miRNA first-strand cDNA synthesis kit (Tiangen). qRT-PCR analysis for mature miRNA was conducted using miRcute miRNA qRCR detection kit (Tiangen). For measurement of miRNA or pre-miRNA levels in the tissue samples,18S rRNA was used as an internal control. The miRNA levels in serum and exosomes were determined using absolute quantitative real-time RT-PCR described previously^40^. Primer sequences for these miRNAs were listed in Table S2.

### Western blotting

After tissues were homogenized in liquid nitrogen, the homogenate was lysed on ice for 30 min in lysis buffer (BioTeKe, Beijing, China). The lysates (20–40 μg) of total protein were loaded per well and separated on a 10% SDS-polyacrylamide gel. Primary antibodies were anti-CTGF (1:5000 dilution, Abcam, Shanghai, China), anti-collagen-II (1:5000 dilution, Abcam), anti-collagen-IV (1:5000 dilution, Abcam), anti-Smad 2/3 (1:1000 dilution, Cell Signaling Technology, Boston, MA), anti-Smad 7 (1:1000 dilution, Santa Cruz Biotech, Santa Cruz, CA), anti-p-Smad2 (1:1000 dilution, Cell Signaling Technology), and anti-actin antibodies (1:5000 dilution, Santa Cruz Biotech). The secondary antibody was a peroxidase-coupled anti-goat IgG (GE Healthcare). The membrane was exposed to ECL Hyperfilm (GE Healthcare), and the film was developed. The bands were quantified by densitometry using ImageJ. Results were from triplicate experiments.

### Functional assessment by echocardiography

Rats fed HFD for 24 weeks and age-matched control rats (n = 6 each experiment) underwent transthoracic echocardiography under anesthesia (pentobarbital sodium, 60 mg/kg ip) in a supine position. The procedure was performed by an expert operator blinded to treatment assignment using a VisualSonics ultrasound machine a Samsung accuvix A30 (Bothell, USA) with an ultrasound probe Medison A30-1. Left ventricular end-diastolic diameter (LVEDD) was measured in M-mode standard two-dimensional (2DE) of long-axis parasternal view as the distance between interventricular septum and LV posterior wall at the time of LVED (widest diameter). Moreover, left ventricular end-systolic diameter (LVESD) was measured as the distance between interventricular septum (IVS) and LV posterior wall (PW) at the time of LVES (narrowest diameter). The LV ejection fraction (EF) and fractional shortening, indices of global systolic function, were calculated as: EF = ((LVEDV − LVESV)/LVEDV × 100, where LVEDV is LV end-diastolic volume and LVSV is LV end-systolic volume; and fractional shortening = ((LVEDD − LVESD)/LVEDD) × 100.

### Microarray analysis

The heart samples, which were collected from 14 rats fed normal rat chow diet and HFD for 24 weeks, respectively, were sent to Kangchen Bio-tech Incorporation (Shanghai, China) for miRNA microarray. Briefly, miRNAs from the heart samples were extracted using miRNeasy mini kit (QIAGEN, Mainz, Germany). After having passed RNA quantity measurement using the NanoDrop 1000, the samples were labeled using the miRCURY™ Hy3™/Hy5™ Power labeling kit and hybridized on the miRCURY™ LNA Array (v.18.0). Following the washing steps the slides were scanned using the Axon GenePix 4000B microarray scanner (Molecular Devices, Sunnyvale, CA). Scanned images were then imported into GenePix Pro 6.0 software (Axon) for grid alignment and data extraction. Replicated miRNAs were averaged and miRNAs that intensities > = 30 in all samples were chosen for calculating normalization factor. Microarray data sets are provided in Table S1. miRNAs with a 2-fold or greater fold change and a *P*-value < 0.05 were considered differentially expressed.

### Cell culture

H9c2 cells (a gift from Dr. Di Lu, Kunming Medical University) were grown in Dulbecco’s Modified Eagle Medium (Hyclone, Logan, UT) supplemented with 10% Fetal Bovine Serum (FBS, Hyclone). When 60–80% confluent, the cells were transfected using Lipofectamine 2000® Reagent (Thermo Fisher Scientific, Waltham, MA) with 0.25 μg of the negative control or rno-miR-410-5p mimics: 5′-Agguugucugugaugaguucg-3′ labeled with 2′-Ome (obtained from GenePharma Company, Shanghai, China). Cells were collected 48 h after transfection, the protein levels of fibrotic genes were determined by western blotting.

### Luciferase assay

To construct reporter vectors bearing miRNA-binding sites, the sequence of 3′-untranslated region (3′UTR) of Smad 7 was amplified from genomic DNA by PCR, and inserted into the multiple cloning sites downstream of the luciferase gene in the psiCHECKTM-2 luciferase miRNA expression reporter vector (Promega, Beijing, China). H9c2 cells were cultured in 24-well plates were transfected using Lipofectamine 2000® Reagent (Thermo Fisher Scientific) with 0.25 μg of the vector as well as rno-miR-410-5p mimics. Cells were collected 48 h after transfection. The luciferase activity was determined using the Dual-Luciferase reporter assay kit (Promega) on a fluorescent microplate reader (Molecular Devices). Results were normalized to the renilla luciferase control.

### Construction of the lentiviral vectors, lentivirus production, and injection

Lentivirus vectors expressing pre-miR-410 and miR-410-5p antimiR were obtained from GenePharma Co. (Shanghai, China). In brief, pre-miR-410 (5′-ACTTGAGGAGAGGTTGTCTGTGATGAGTTCGCTTT ATTAATGACGAATA TAACACAGATGGCCTGTTTTCAATACC-3′) was inserted into the lentivirus vector PGLV3/H1/GFP + Puor vector (PGLV3), resulting in the vector PGLV3-pre-miR-410. miR-410-5p antimiR (5′-CGAACUCAUCAGAGACAACC-3′) was inserted into the lentivirus vector PGLV3/H1/GFP + Puor vector (PGLV3), resulting in the vector PGLV3-miR-410-5p antimiR. As a negative control, a DNA fragment (5′-TTCTCCGAACGTGTCACGT-3′) was inserted into the PGLV3/H1/GFP + Puor vector, resulting in the vector PGLV3-NC.

The rats were infected with lentivirus particles containing PGLV3-miR-410 or PGLV3-miR-410-5p antimiR or PGLV3-NC was co-transfected with three other helper plasmids (PG-P1-Gag- VSVG, PG-P2-REV, and PG-P3-RRE) into human embryonic kidney 293 T cells. The supernatant was collected at 48 h post-transfection, and fresh medium was added to the culture flask. The cells were cultured for another 24 h. Then, the supernatant was collected again. The virus vector-containing supernatants collected from 48 and 72 h were mixed. The mixture was centrifuged at 3000 rpm for 15 min at 4 °C. Then the liquid was filtered by a 0.45-mm filter membrane. The acquired virus was stored at −80 °C until use. The titers of the PGLV3-miR-410 or PGLV3-NC vectors were 1 × 10^9^ transfer units (TU/ml).

Twelve-week-old rats fed a standard laboratory chow were infected with 1 × 10^8^ TU of lentivirus particles containing empty vector PGLV3 or vector expressing pre-miR-410 through tail vein injection. Injection of these lentivirus particles was repeated twice for the next two weeks in rats. Rats were sacrificed at day 21 after the first injection to collect the heart samples.

Rats fed HFD for 21 weeks were injected with 1 × 10^8^ TU of lentivirus particles containing PGLV3-miR-410-5p antimiR or PGLV3-NC through tail vein injection. Injection of these lentivirus particles was repeated twice for the next two weeks in rats fed HFD. Rats were sacrificed at day 21 after the first injection to collect the heart samples.

### Construction of the adenovirus vectors, adenovirus production, and injection

Adenovirus vectors for adipocyte- or kidney-specific overexpression of pre-miR-410 were obtained from Cyagen Co. (Guangzhou, Guangdong, China). For adipocyte-specific overexpression, pre-miR-410 was inserted into the adenovirus vector pAV[Exp]-aP2 > EGFP containing the promoter of adipocyte-specific gene aP2/FABP4, resulting in the vector pAV[Exp]-aP2 > EGFP:{rmiR-410}. For kidney-specific overexpression, pre-miR-410 was inserted was inserted into the adenovirus vector pAAV[Exp]-NPHS2 > EGFP containing the promoter of kidney-specific gene NPHS2/Podocin, resulting in the vector pAV[Exp]- NPHS2 > EGFP:{rmiR-410}. Details of the adenovirus vectors can be found at https://en.vectorbuilder.com/vector/VB171102-1059rjs.html and https://en.vectorbuilder.com/vector/VB171103-1023hst.html.

These shuttle plasmids were co-transfected into HEK-293T human embryonic kidney 293A cells with AAV-DJ/8 plasmid (Cell Biolabs Inc., San Diego, CA) and pHelper plasmid (Cell Biolabs) for virus packaging. The cells and medium were collected after the cytopathic effect was apparent. After three cycles of freeze and thaw to release the virus, the cell debris was discarded by centrifugation at 3,000 g × 10 min. The virus-containing supernatant was purified using CsCl density gradient centrifugation, followed by dialysis against saline. The viral titration was determined and adjusted to 1 × 10^10^ PFU/ml.

Rats fed normal diet were infected with 5 × 10^8^ TU of adenovirus particles containing empty vector or vector expressing pre-miR-410 through tail vein injection. Injection of these virus particles was repeated twice for the next two weeks in rats fed normal diet. Rats were sacrificed at day 21 after the first injection to collect the heart samples.

### Statistical analysis

Data from experiments are expressed as means ± SD of three independent experiments. Statistical differences between two groups were analyzed using Student’s t-test. Values of *P* < 0.05 were considered statistically significant.

## Electronic supplementary material

Supplementary Information 
