# Supplementary material for: MicroRNA-410-5p exacerbates high-fat diet-induced cardiac remodeling in mice in an endocrine fashion
Source: Sci Rep. 2018 Jun 8;8:8780. doi: 10.1038/s41598-018-26646-4 (PMC5993721; doi:10.1038/s41598-018-26646-4)
Supplement: Supplementary file 1 — Supplementary Information [file 41598_2018_26646_MOESM1_ESM.docx]

**Supplemental Information for**

**MicroRNA-410-5p exacerbates high-fat diet-induced cardiac remodeling in mice in an endocrine fashion**

Tong Zou, Mei Zhu, Yi-Cheng Ma, Fei Xiao, Xue Yu, Li Xu, Lan-Qing Ma, Jiefu Yang, Jian-Zeng Dong


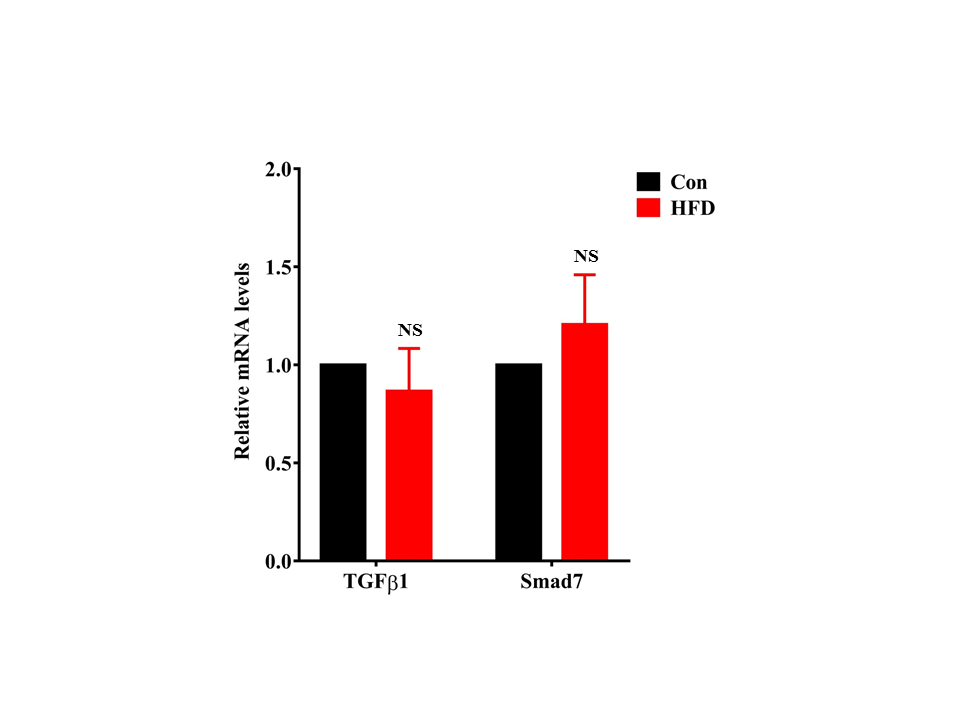


**Figure S1 HFD does to influence the mRNA levels of TGFβ1 and Smad 7.**

The mRNA levels of TGFβ1 and Smad 7 were detected by qPCR in the heart of rats fed normal diet and HFD for 24 weeks (n=10 in each experiment). NS, not statistically significant.


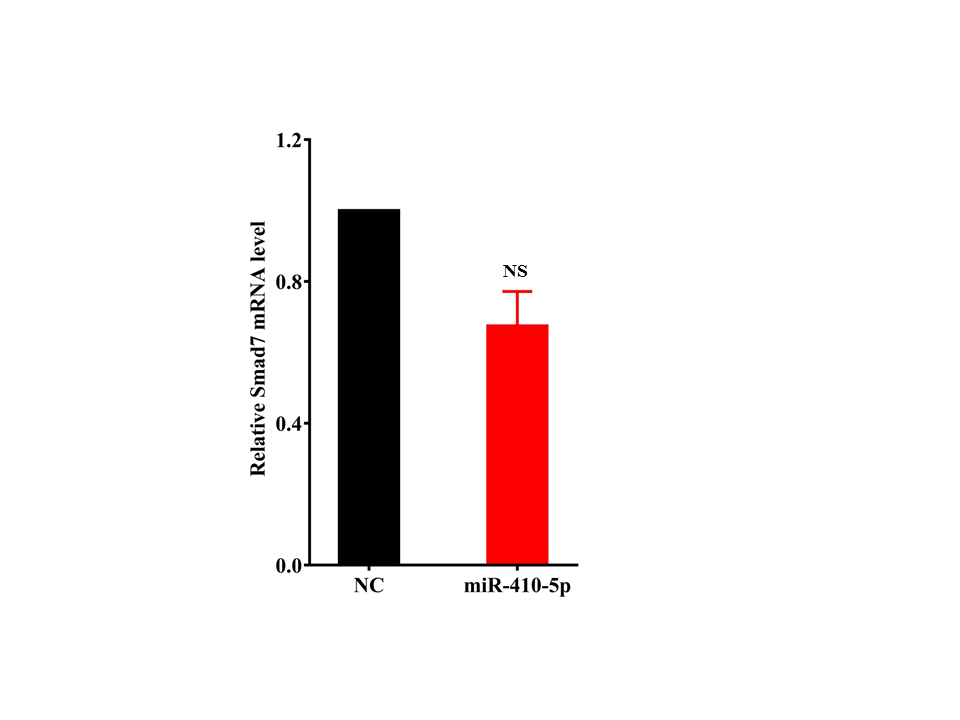


**Figure S2** **Overexpression of miR-410-5p does not influence the mRNA expression of Smad 7.**

The rats were infected with lentivirus particles containing PGLV3-miR-410 or PGLV3-NC. The mRNA levels of Smad 7 in the heart of rats were detected by qPCR (n=6 in each experiment). NS, not statistically significant.


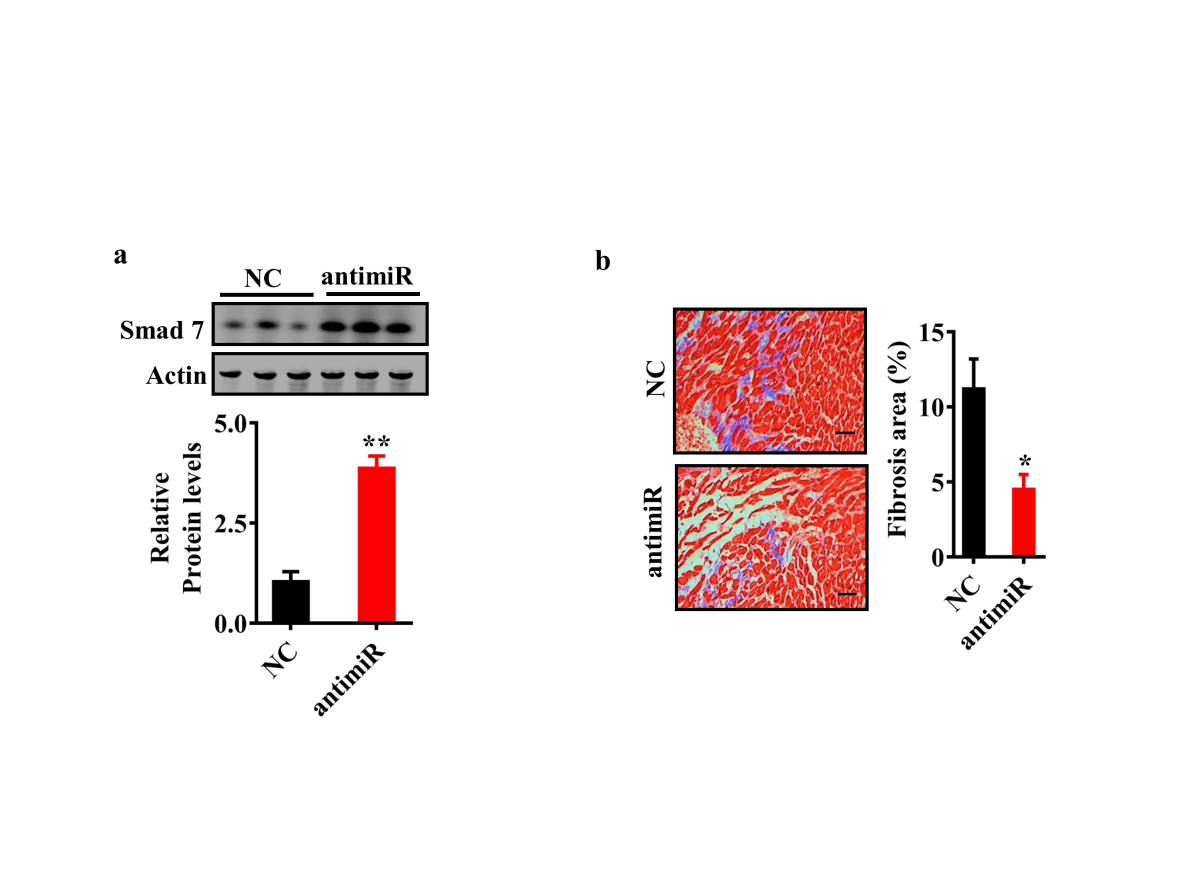


**Figure S3 Inhibition of miR-410-5p by miR-410-5p antimiR attenuates cardiac fibrosis in rats fed HFD.**

(a)The protein levels of Smad 7 were measured using Western blotting. Representative Western blots are shown. Quantification of the ratio of proteins to β-actin (Lower panel). These results are the means ± SD of three experiments (n =6 in each experiment). **P< 0.01 relative to negative control (NC). (b) Masson’s staining of hearts in rats. Quantitative analysis of fibrotic area (Right panel). These results are means±SD of three experiments (n=10 in each experiment). *P< 0.05 relative to negative control (NC).


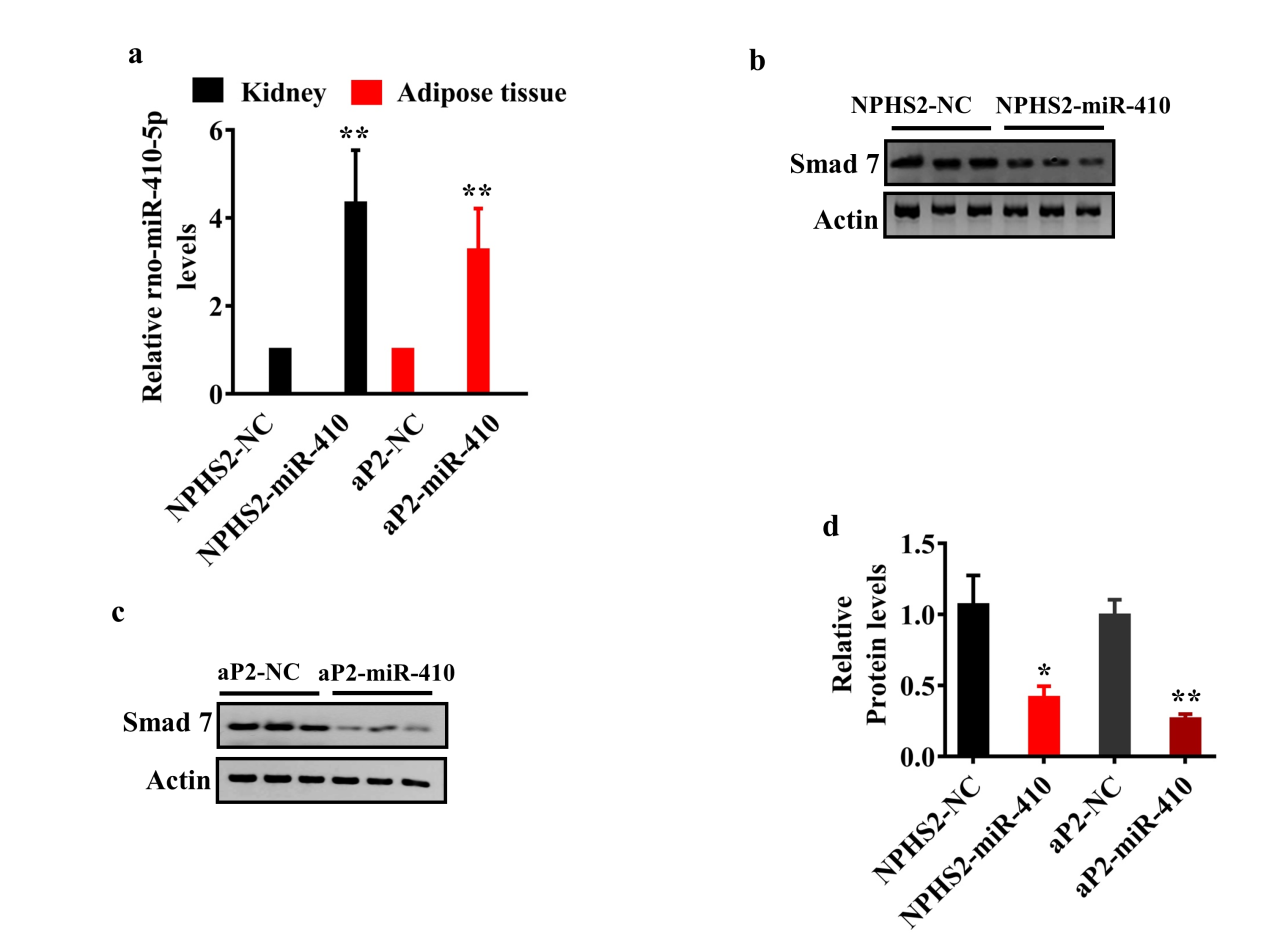


**Figure S4 Kidney- or adipocyte-specific overexpression of miR-410 inhibits Smad 7 protein levels.**

(a) The mRNA levels of miR-410-5p were detected by qPCR in the kidney or adipose tissue with kidney- or adipocyte-specific overexpression of miR-410 (n=6 in each experiment). NPHS2-miR-410, kidney-specific overexpression; aP2-miR-410, adipocyte-specific overexpression. **P< 0.01 relative to negative control (NC). (b and c) The protein levels of Smad 7 were measured using Western blotting in the heart of rats fed normal diet with (b) kidney- or (c) adipocyte-specific overexpression of miR-410. Representative Western blots are shown. (d) Quantification of the ratio of proteins to β-actin. These results are the means ± SD of three experiments (n=6 in each experiment). *P< 0.05;**P< 0.01 relative to negative control (NC).


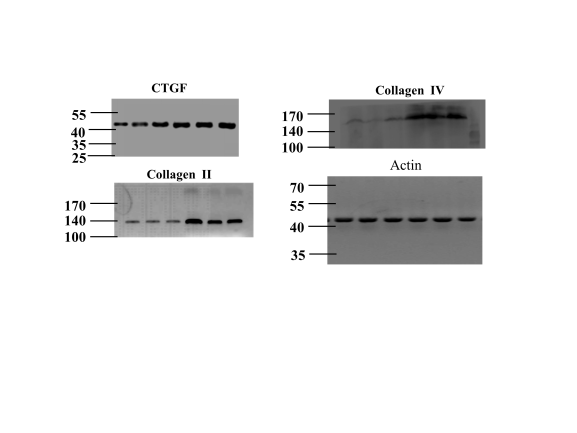

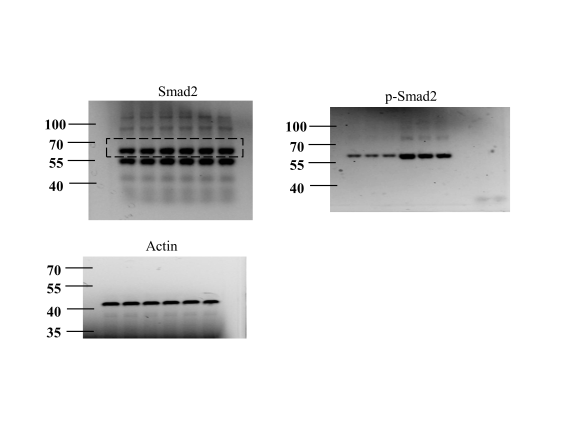


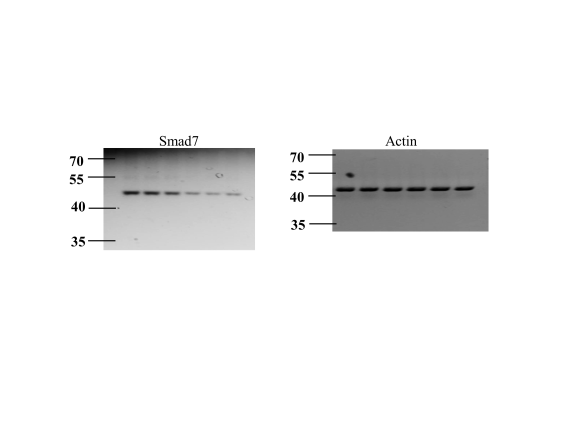

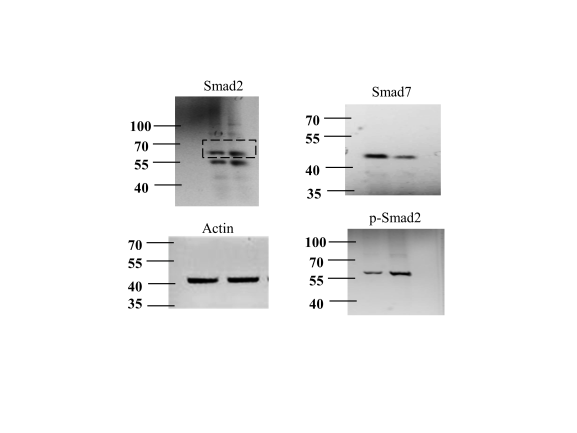


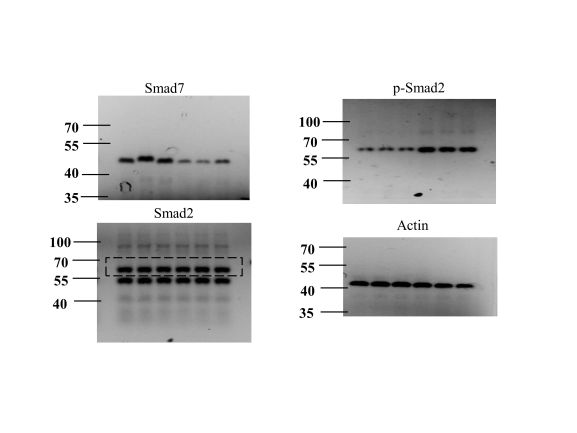

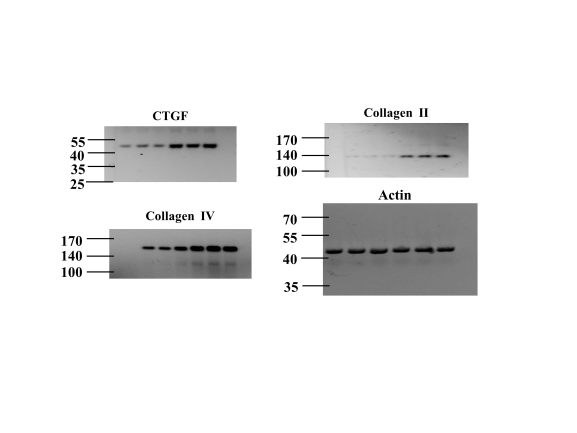


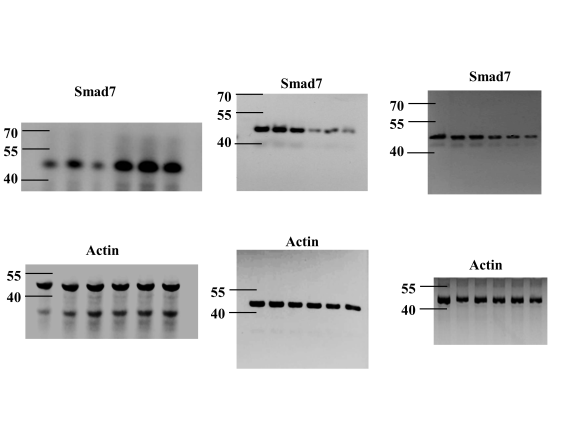


**Figure S5 Full length of Western blots.**

**Table S1 miRNA expression in the heart of mice fed HFD.**

| **ID** | **Name** | **Fold change**  **(HFD vs Con)** | ***P*-value** |
| --- | --- | --- | --- |
| 148135 | rno-miR-3583-3p | 7.74816444 | 0.034797519 |
| 148187 | rno-miR-410-5p | 7.669580257 | 0.01315213 |
| 42849 | rno-miR-146b-3p | 6.73981324 | 0.030487341 |
| 29779 | rno-miR-764-5p | 6.460950419 | 0.018539003 |
| 145843 | rno-miR-330-5p | 5.155791221 | 0.037676781 |
| 145708 | rno-miR-324-3p | 5.15024782 | 0.001966311 |
| 145947 | rno-miR-301a-5p | 4.787760101 | 0.045848283 |
| 42938 | rno-miR-343 | 4.746072159 | 0.000297526 |
| 148627 | rno-miR-615 | 4.549157633 | 0.032574535 |
| 42453 | rno-miR-376c-5p | 4.50083076 | 0.00951711 |
| 148455 | rno-miR-741-3p | 4.05067599 | 0.03404348 |
| 10937 | rno-miR-132-3p | 3.751644383 | 0.001465713 |
| 31053 | rno-miR-674-3p | 3.305763842 | 0.004286871 |
| 145640 | rno-miR-328a-3p | 3.224539169 | 0.000187177 |
| 13178 | rno-miR-18a-3p | 3.079446718 | 0.022407757 |
| 42862 | rno-miR-881-3p | 3.044041217 | 0.005934456 |
| 145820 | rno-let-7c-5p | 3.02129627 | 0.01498634 |
| 148482 | rno-miR-874-5p | 3.017293056 | 0.015418576 |
| 145753 | rno-miR-484 | 3.008357552 | 0.024148242 |
| 148360 | rno-miR-375-5p | 2.990715985 | 0.001010111 |
| 33596 | rno-miR-126a-5p | 2.918802652 | 0.017095675 |
| 148459 | rno-miR-3593-3p | 2.885134742 | 5.79523E-05 |
| 148488 | rno-miR-3590-5p | 2.796128886 | 6.47553E-06 |
| 148126 | rno-miR-3550 | 2.735488704 | 0.001892261 |
| 146163 | rno-miR-224-3p | 2.667888201 | 0.002118741 |
| 145970 | rno-miR-129-2-3p | 2.543151888 | 0.008018469 |
| 13150 | rno-miR-322-5p | 2.487788814 | 0.031022115 |
| 42769 | rno-let-7b-3p | 2.486019543 | 0.043131839 |
| 168890 | rno-miR-1306-5p | 2.462404468 | 0.011050702 |
| 146137 | rno-miR-133a-3p | 2.418583717 | 0.000606586 |
| 146160 | rno-miR-133b-3p | 2.37276317 | 0.000175534 |
| 148069 | rno-miR-129-1-3p/rno-miR-129-2-3p | 2.365903502 | 0.020256917 |
| 148348 | rno-miR-3573-3p | 2.350561417 | 9.34824E-05 |
| 148456 | rno-miR-3547 | 2.316153861 | 0.000277017 |
| 11247 | rno-miR-434-5p | 2.297494813 | 0.016625884 |
| 148271 | rno-miR-328b-3p | 2.266355994 | 0.00452041 |
| 145897 | rno-miR-92b-3p | 2.26167906 | 0.002281352 |
| 148584 | rno-miR-3572 | 2.228168142 | 1.12465E-06 |
| 147198 | rno-miR-26a-5p | 2.215380998 | 0.013315309 |
| 148404 | rno-miR-299b-5p | 2.212715297 | 0.029087953 |
| 146038 | rno-miR-504 | 2.198501302 | 6.2967E-05 |
| 148294 | rno-miR-217-3p | 2.190200815 | 0.034600452 |
| 148359 | rno-miR-3596d | 2.109913339 | 0.007925489 |
| 168830 | rno-miR-148b-5p | 2.107231614 | 0.04090416 |
| 148315 | rno-miR-3594-3p | 2.091822938 | 0.006589316 |
| 148583 | rno-miR-3584-3p | 2.074997667 | 4.41963E-05 |
| 148257 | rno-miR-3546 | 2.032564708 | 0.009752318 |
| 10925 | rno-miR-10b-5p | 2.025576672 | 0.002780659 |
| 145840 | rno-let-7f-1-3p | 2.021060927 | 1.15467E-05 |
| 42538 | rno-miR-196a-3p | 2.007579793 | 0.007194132 |
| 42808 | rno-miR-874-3p | 0.496012976 | 0.018293937 |
| 46251 | rno-miR-1193-3p | 0.48250345 | 0.018152696 |
| 11020 | rno-miR-22-3p | 0.477564899 | 0.024582475 |
| 42502 | rno-miR-204-3p | 0.477039403 | 0.000332446 |
| 10977 | rno-miR-183-5p | 0.47520165 | 0.043672208 |
| 148477 | rno-miR-1843-5p | 0.474365428 | 0.001439789 |
| 17953 | rno-miR-183-3p | 0.46954784 | 0.019079466 |
| 42826 | rno-miR-300-5p | 0.455353476 | 0.046792413 |
| 17898 | rno-miR-99b-3p | 0.454827087 | 0.006532304 |
| 10943 | rno-miR-136-5p | 0.444758468 | 0.011832775 |
| 11262 | rno-miR-297 | 0.441791375 | 0.004842974 |
| 10986 | rno-miR-193-3p | 0.431611833 | 0.008630937 |
| 148192 | rno-miR-421-3p | 0.430143144 | 0.001148267 |
| 19585 | rno-miR-148b-3p | 0.427207796 | 0.014611215 |
| 42630 | rno-miR-140-3p | 0.426880571 | 0.006376171 |
| 17896 | rno-miR-21-3p | 0.42571657 | 0.023149733 |
| 29490 | rno-miR-7a-5p | 0.425429179 | 0.000234573 |
| 145634 | rno-miR-132-5p | 0.422843772 | 0.040348202 |
| 10998 | rno-miR-19b-3p | 0.422085165 | 0.038907304 |
| 11074 | rno-miR-34c-5p | 0.414050018 | 0.002380369 |
| 11041 | rno-miR-29c-3p | 0.400911629 | 0.002379242 |
| 10947 | rno-miR-142-3p | 0.3895185 | 0.028257299 |
| 10988 | rno-miR-194-5p | 0.377650957 | 0.020465994 |
| 148150 | rno-miR-3559-5p | 0.373472587 | 0.001449171 |
| 4610 | rno-miR-126a-3p | 0.372859568 | 0.013433678 |
| 145968 | rno-let-7d-5p | 0.351071088 | 0.003719445 |
| 42866 | rno-miR-451-5p | 0.348760581 | 0.000135045 |
| 148397 | rno-miR-664-1-5p | 0.339479085 | 0.02404667 |
| 148284 | rno-miR-208b-3p | 0.336817612 | 0.026753632 |
| 18739 | rno-miR-186-5p | 0.323844875 | 0.032332546 |
| 32884 | rno-miR-342-3p | 0.315177687 | 0.026657124 |
| 148053 | rno-miR-667-5p | 0.310874323 | 0.030146174 |
| 42490 | rno-miR-505-5p | 0.307322977 | 0.000312335 |
| 148174 | rno-miR-653-3p | 0.294863592 | 0.039032606 |
| 145676 | rno-miR-30e-3p | 0.284969927 | 0.002207118 |
| 169132 | rno-miR-382-3p | 0.276245003 | 0.002655193 |
| 148291 | rno-miR-496-5p | 0.263920015 | 0.049955464 |
| 13148 | rno-miR-195-5p | 0.24869745 | 0.004236953 |
| 10995 | rno-miR-199a-3p | 0.223386323 | 0.005167783 |
| 28250 | rno-miR-872-5p | 0.210403634 | 0.029596565 |
| 42933 | rno-miR-466b-5p | 0.183713731 | 9.70996E-05 |
| 148260 | rno-miR-664-2-5p | 0.154999587 | 0.002736587 |
| 145943 | rno-miR-100-5p | 0.1292468 | 0.030291888 |
| 11052 | rno-miR-31a-5p | 0.084599888 | 9.6631E-06 |

**Table S2 Primer sequences.**

| **Gene** | **Forward Primer** | **Reverse Primer** |
| --- | --- | --- |
| CTGF | ACTATGATGCGAGCCAACTGC | TGTCCGGATGCACTTTTTGC |
| Collagen I | TGGCCTTGGAGGAAACTTTTG | CTTGGAAACCTTGTGGACCAG |
| Collagen II | GATGGCTCTAATGGAATCCCTG | GCTGTCTCAAGGTACTGTCTG |
| Collagen III | TTGAATATCAAACACGCAAGGC | GGTCACTTTCACTGGTTGACGA |
| Collagen IV | GTGAAATTCTCGGCCATGTTC | TCTCCCTTGTCGCCTTTTG |
| TGFβ1 | CCAAGGAGACGGAATACAGG | GTGTTGGTTGTAGAGGGCAAG |
| GAPDH | GATGGGTGTGAACCACGAGAAA | ACGGATACATTGGGGGTAGGAA |
| Pre-rno-miR-410 | AGGTTGTCTGTGATGAGTTCG | AGGCCATCTGTGTTATATTCGT |
| 18S rRNA | TGTTCACCATGAGGCTGAGATC | TGGTTGCCTGGGAAAATCC |
